# Effects of arbuscular mycorrhizal fungi and soil nutrient addition on the growth of *Phragmites australis* under different drying-rewetting cycles

**DOI:** 10.1371/journal.pone.0191999

**Published:** 2018-01-29

**Authors:** Jin-Feng Liang, Jing An, Jun-Qin Gao, Xiao-Ya Zhang, Fei-Hai Yu

**Affiliations:** 1 School of Nature Conservation, Beijing Forestry University, Beijing, China; 2 Zhejiang Provincial Key Laboratory of Evolutionary Ecology and Conservation, Taizhou University, Taizhou, China; Shandong University, CHINA

## Abstract

The frequency of soil drying–rewetting cycles is predicted to increase under future global climate change, and arbuscular mycorrhizal fungi (AMF) are symbiotic with most plants. However, it remains unknown how AMF affect plant growth under different frequencies of soil drying–rewetting cycles. We subjected a clonal wetland plant *Phragmites australis* to three frequencies of drying-rewetting cycles (1, 2, or 4 cycles), two nutrient treatments (with or without), and two AMF treatments (with or without) for 64 days. AMF promoted the growth of *P*. *australis*, especially in the 2 cycles of the drying-rewetting treatment. AMF had a significant positive effect on leaf mass and number of ramets in the 2 cycles of the drying-rewetting treatment with nutrient addition. In the 2 cycles of drying-rewetting treatment without nutrient addition, AMF increased leaf area and decreased belowground to aboveground biomass ratio. These results indicate that AMF may assist *P*. *australis* in coping with medium frequency of drying-rewetting cycles, and provide theoretical guidance for predicting how wetland plants respond to future global climate change.

## Introduction

Current climate models predict more intense rainstorms and extreme drought events under future global climate change [1]. The increase in evapotranspiration and soil drought will increase the frequency of soil drying–rewetting cycles [2]. This would negatively affect wetlands, which are regarded as one of the ecosystems most sensitive to future changes [3]. Wetlands are one of the most important and productive ecosystems on earth [4], with high economic, cultural, and recreational value [5]. Wetland plants play an important role in ecological function of wetlands and are a critical component of wetland ecosystems [6,7]. Therefore, how wetland plants respond to the frequency of soil drying–rewetting cycles is important to predict the potential impact of future global climate change on wetland ecosystems [5].

Arbuscular mycorrhizal fungi (AMF) form symbiotic association with the roots of most terrestrial plants [8]. Historically, AMF were thought to be absent or rare in wetlands, due to their low survival possibility in anaerobic conditions [9]. However, increasing evidence demonstrates that AMF can survive in wetland ecosystems and many wetland plants are associated with AMF [10]. Numerous studies have tested the effects of AMF on terrestrial plants [11–14]. These studies generally show that AMF can increase the growth of terrestrial plants by promoting nutrient uptake, especially under nutrient-poor conditions [11,12], although a neutral or negative effect of AMF on plant growth is also reported [13,14]. However, relative few studies have examined effects of AMF on the growth of wetland plants [15–18]. Existing studies on wetland plants showed that AMF colonization increased biomass of *Polygonum cuspidatum* [15], increased shoot/root ratio of *Eclipta prostrata* [18], and had neutral or negative effects on the growth of *P*. *japonica* [15]. AMF can also significantly promote biomass and nutrient uptake of the mangrove species *Kandelia obovata* and *Sonneratia apetala* [16,17]. However, to our knowledge, no study has tested effects of AMF on plant growth under different levels of drying-rewetting cycles.

Many wetlands are subjected to eutrophication due to the input of excess nitrogen (N) and phosphorus (P), arising from fossil fuel combustion, agricultural activities and livestock waste [19,20]. AMF and nutrients could exert an interactive effect on plant growth [21,22]. Under nutrient-poor conditions, host plants may benefit more from the increased uptake of soil nutrients by their fungal symbionts [23]. By contrast, AMF may have a negative effect on host plants under nutrient-rich conditions because AMF take carbohydrates from host plants [24–26]. Therefore, it is also important to examine effects of AMF on wetland plants under different nutrient conditions [27], especially when these wetland plants are subjected to different drying-rewetting cycles.

We grew a common wetland plant *Phragmites australis* under three frequencies of drying-rewetting cycles (1, 2 and 4 cycles), two nutrient addition treatments (with or without) and two AMF treatments (with or without). Specifically, we addressed the following questions: (1) Do AMF affect the growth of the wetland plant *P*. *australis*? (2) Do effects of AMF on the growth of *P*. *australis* depend on the nutrient level and the frequency of drying-rewetting cycles?

## Materials and methods

### Plant species and AMF

The common reed (*Phragmites australis* (Cav.) Trin. ex Steudel) is a clonal perennial plant that grows in a wide variety of ecosystems, including swamp, coastal marshes, inland lakes, and rivers [28, 29]. It has high adaptability, and a well-developed aerenchym and rhizosphere to facilitate colonization, and readily propagates by rhizome or stem node [30, 31] It has a well-developed aerated tissue [32]. Previous studies have showed that *P*. *australis* is a common mycorrhizal plant with an infection rate of more than 30% [31–33].

Rhizomes of *P*. *australis* were collected from Shahe in Beijing, China. They were vegetatively cultivated in a greenhouse in Forest Science Co., Ltd., of Beijing Forestry University in Beijing. After one month, 288 ramets each consisting of a shoot about 15 cm tall and some roots were selected for the experiment. The roots of each ramet were surface-sterilized by 75% ethyl alcohol for 10 seconds and 1% sodium hypochlorite (NaClO) for 15 minutes and then washed by sterile distilled water three times before being transplanted into the sterilized pots.

AMF were obtained from the rhizospheres of *P*. *australis* growing in the same riparian zone in suburban areas of Beijing, China. AMF inocula consists of spores, extraradical mycelium and fine colonized root segments from cultures that were propagated in a sterilized mixture of soil and sand with host sorghum (*Sorghum propinquum* H.) in a glasshouse for three months. Both soil and sand were sieved through a 2-mm mesh before being thoroughly mixed at a volume ratio of 1:1. The soil-sand mixture was then autoclaved at 121°C for 2 h before potting. The field studies did not involve endangered or protected species and no specific permits were required for the described studies.

### Experimental design

The experiment used a factorial design with two levels of AMF (with vs. without), three frequencies of drying-rewetting cycles (1, 2, or 4), and two levels of nutrient addition (with vs without), making a total 12 treatments. Each treatment had six replicates. Four ramets of *P*. *australis* were transplanted into each pot (15 cm in diameter and 18 cm in height), and one uniform ramet was retained after 2 weeks. In the treatment with AMF, we added 50 g fresh inoculum around the roots of the ramet in the pot. In the treatment without AMF, we added 50 g sterilized inoculum and also 10 ml of a suspension of the AMF inoculum, filtered to remove AMF spores but not other smaller soil microorganisms [34,35]. For the treatment with nutrient addition, we added a solution of (NH_4_)_2_HPO_4_ at 90 g·m^-2^·y, and for the treatment without nutrient addition we added distilled water with the same amount.

We applied three drying–rewetting cycle treatments during the 64 days of the experiment. The 1-cycle treatment was the control (CK), in which pots were watered every 2 days to maintain constant moisture; the volumetric water content of soil was maintained at about 17%. In the 2 and 4-cycle treatments, pots were subjected to two or four drying–rewetting events (soil water content ranged between 16.6–21.5%), respectively. One drying-rewetting event included 8 days of dry and 8 days of wet. In the 2-cycle treatment pots encountered two drying–rewetting events at the first and third period of the experiment. In the 4-cycle treatment pots received four drying–rewetting event at all four periods.

The experiment started on 5 August 2016 and ended on 9 October 2016. It was conducted in a greenhouse at Beijing Forestry University under natural light conditions. During the experiment, the mean air temperature ranged between 19.7°C and 27.2°C.

### Measurements

Before harvest, we counted the number of ramets and measured ramet height of *P*. *australis* in each pot. Then we harvested leaves, stems, and belowground parts (roots and rhizomes) of *P*. *australis* in each pot separately. Leaves were scanned using an Epson perfection v700 photo scanner, and leaf area was then measured using Image J. After that, we measured dry mass of all plant parts after oven-drying them at 70°C for 48 h. Samples of fresh roots were taken from each plant and cut into approximately 1-cm pieces to determine the colonization rate of AMF. Root samples were cleaned in 10% KOH (w/v) and stained using lactic acid fuchsin solution [36]. The root colonization rates were measured using the gridline-intersect method under a microscope [37].

### Statistical analysis

Before analyses, the data on growth were checked for normality and homogeneity of variance, and no data transformation was used. We used three-way ANOVA to examine the effects of AMF, drying-rewetting cycles, and nutrient addition on biomass, number of ramets, leaf area, plant height and belowground to aboveground biomass ratio of *P*. *australis* and the colonization rates of AMF on roots. We used Tukey HSD tests for multiple comparisons. All the statistical analyses were conducted using SPSS 18.0 (SPSS, Chicago, IL, USA).

## Results

**Fig 1:**
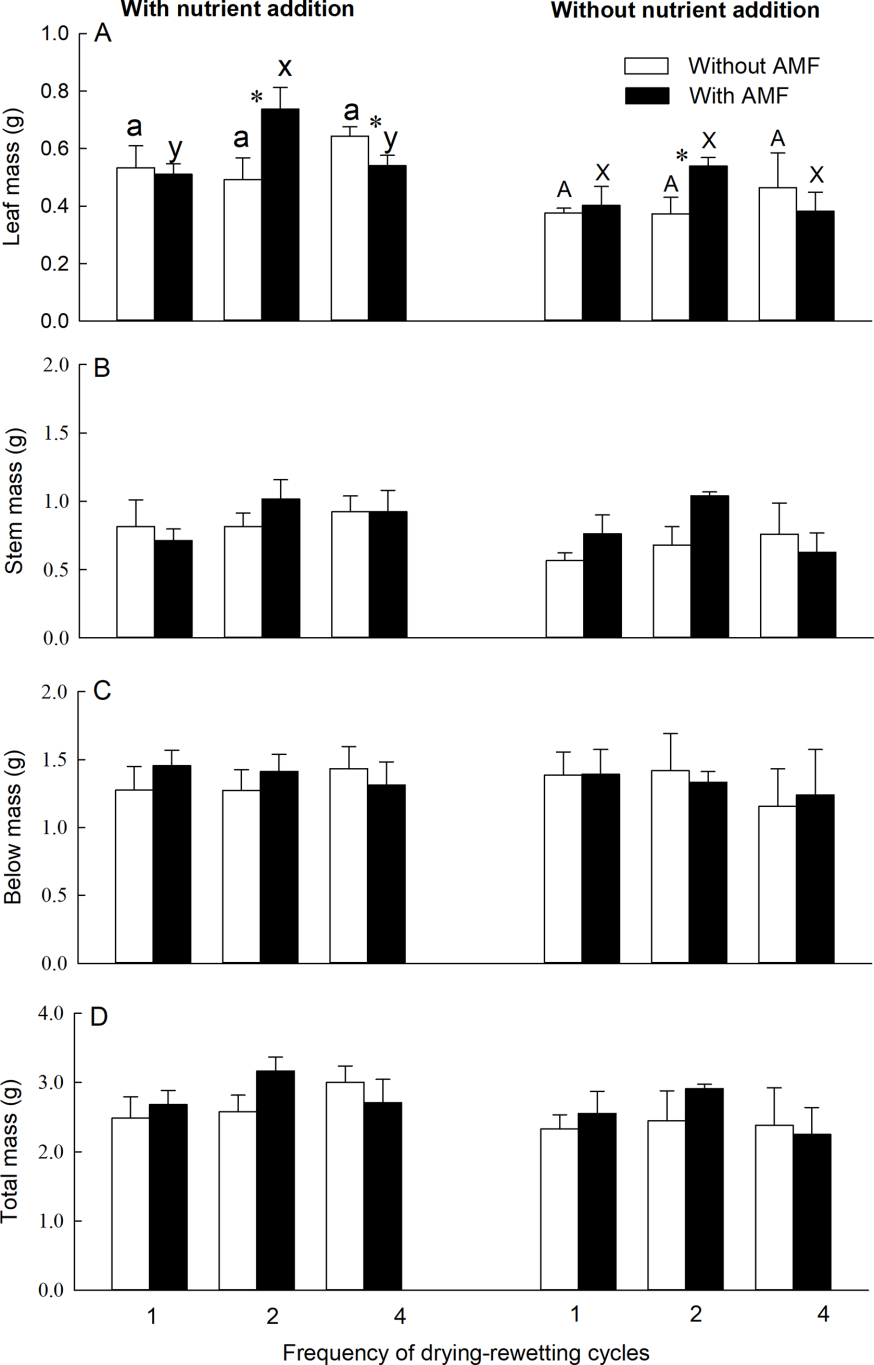
Effects of AMF, drying-rewetting cycles, and nutrient addition on biomass of *Phragmites australis*. Bars and error bars show means and SE (n = 6). 1 = control (one drying-rewetting cycles); 2 = two drying-rewetting cycles; 4 = four drying-rewetting cycles. Within each AMF treatment, bars sharing the same letter are not significantly different at *P* = 0.05. Asterisks show that means differ significantly between the two treatments with and without AMF.

Leaf mass of *P*. *australis* was significantly affected by nutrient addition (S1 Table) and the interaction between AMF and drying-rewetting cycles (F_2, 60_ = 5.86, *P* = 0.005, S1 Table). With AMF and nutrient addition, leaf mass was significantly higher in the 2-cycle than in the 1- and 4-cycle treatment (Fig 1A). Compared to the absence of AMF, the presence of AMF increased leaf mass of *P*. *australis* both with and without nutrient addition in the 2-cycle treatment (Fig 1A). However, AMF decreased leaf mass with nutrient addition under the 4-cycle treatment (Fig 1A).

There was no significant main effect of AMF, or drying-rewetting cycles on the leaf, stem, belowground mass, or total biomass of *P*. *australis* (S1 Table). There was no significant interactive effect of AMF × nutrient addition or nutrient addition × drying-rewetting cycles on leaf, stem, belowground, or total biomass of *P*. *australis* (S1 Table).

**Fig 2:**
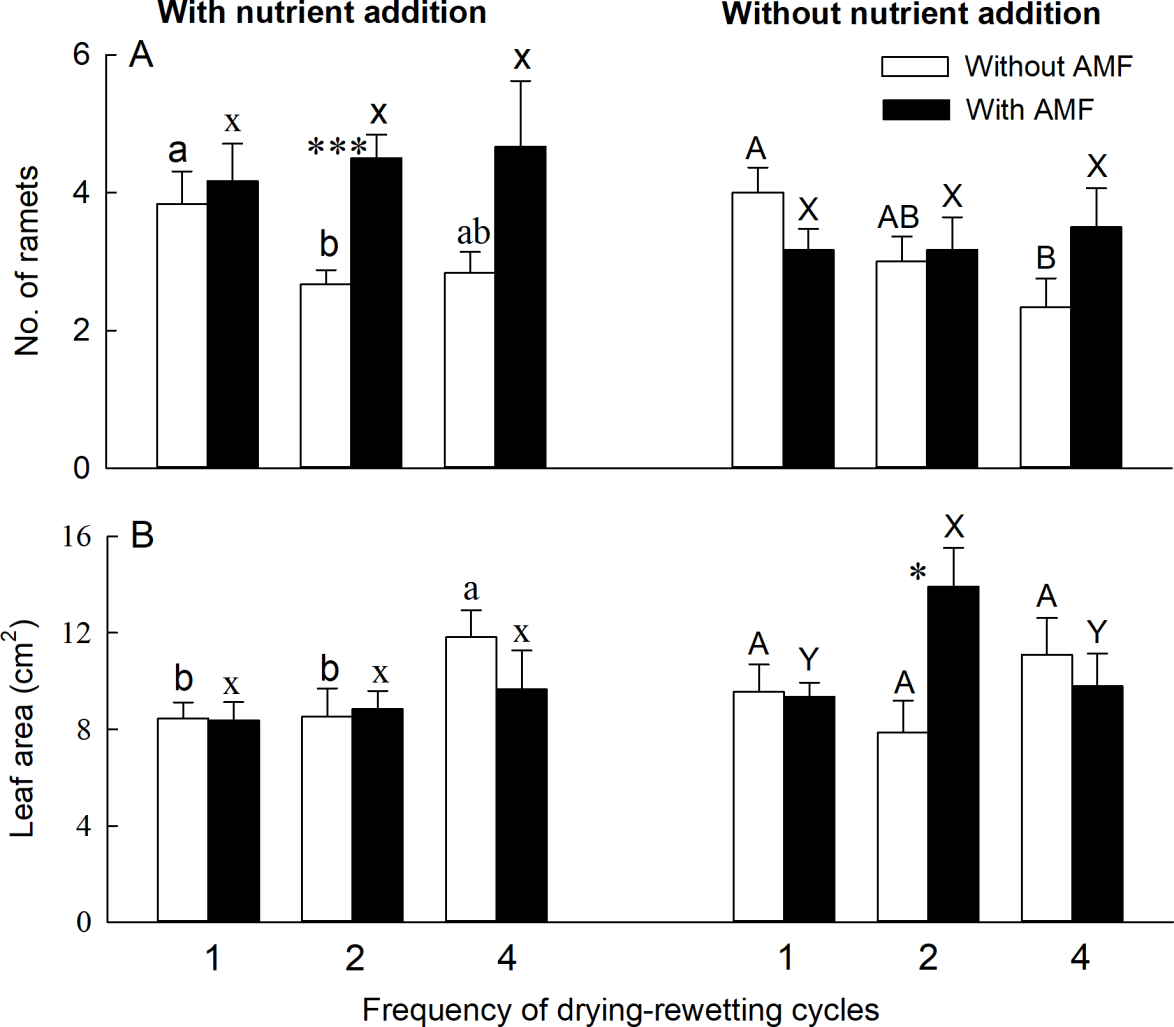
Effects of AMF, drying-rewetting cycles, and nutrient addition on number of ramets and leaf area of *Phragmites australis*. Bars and error bars show means and SE, (n = 6). Treatment codes are described as in Fig 1. Within each AMF treatment, bars sharing the same letter are not significantly different at *P* = 0.05. Asterisks show that means differ significantly between the treatments with and without AMF.

**Fig 3:**
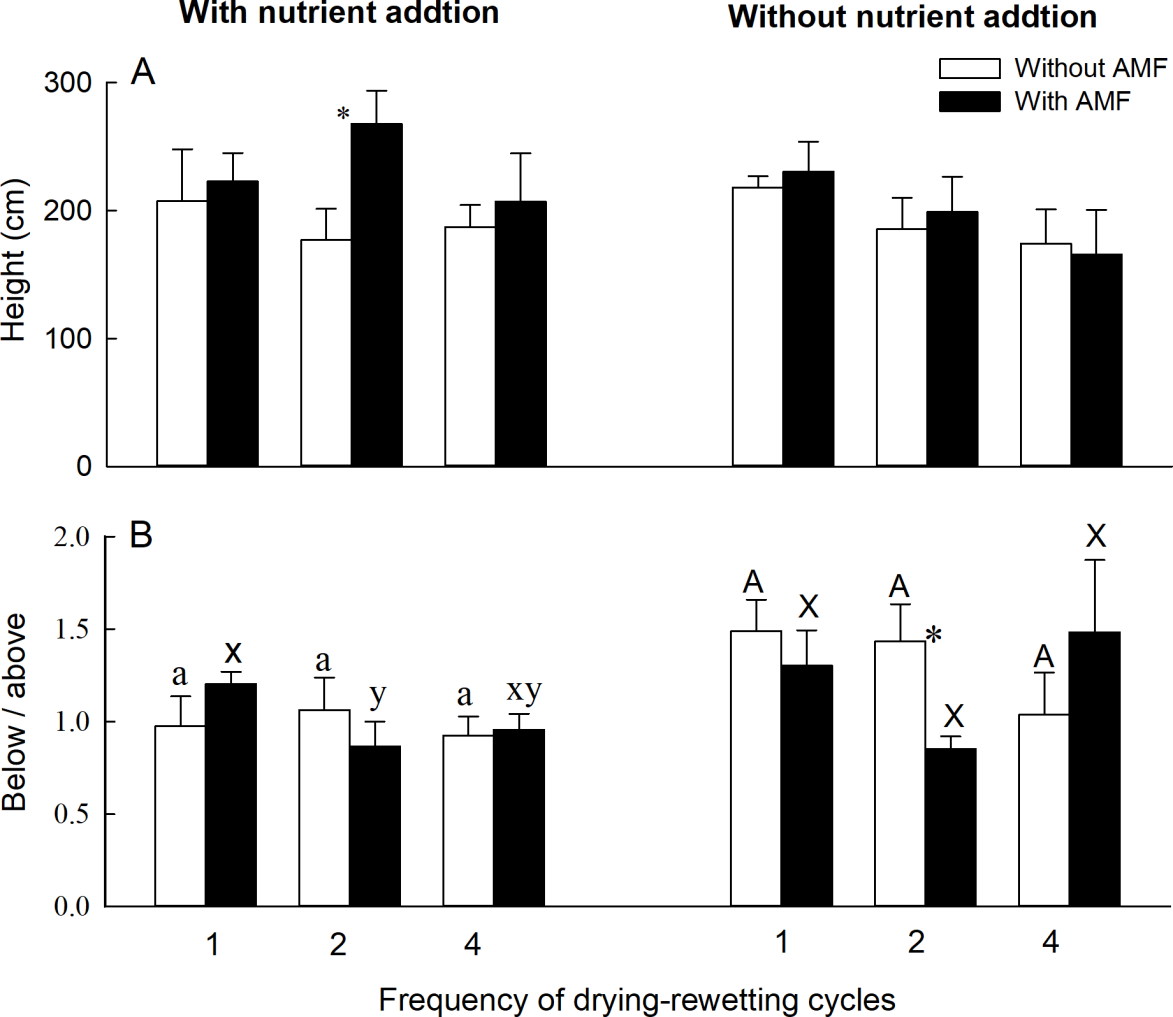
Effects of AMF, drying-rewetting cycles, and nutrient addition on height and below/above of *Phragmites australis*. Bars and error bars show means and SE (n = 6). Treatment codes are described as in Fig 1. Within each AMF treatment, bars sharing the same letter are not significantly different at *P* = 0.05. Asterisks show that means differ significantly between the treatments with and without AMF.

AMF significantly affected the number of ramets (S2 Table), and nutrient addition significantly affected both the number of ramets and ramet height (S2 Table). The number of ramets was significantly higher with than without AMF in the 2-cycle treatment with nutrient addition (Fig 2A). Drying-rewetting cycles significantly affected belowground to aboveground biomass ratio (S2 Table). Belowground to aboveground biomass ratio was significantly lower with than without AMF in the 2-cycle treatment without nutrient addition (Fig 3B).

There was a significant interactive effect of AMF and drying-rewetting cycles on the number of ramets and leaf area (S2 Table). There was also a significant interactive effect between AMF and nutrient addition on the number of ramets (S2 Table). Leaf area was significantly higher with AMF than without AMF in the 2-cycle treatment without nutrient addition (Fig 2B). There was no significant effect of AMF on stem mass, belowground mass or total mass of *P*. *australis*.

The number of ramets was not significantly affected by any of the treatments with AMF (Fig 2A). Leaf area of *P*. *australis* was significantly higher in the 2-cycle than 1- and 4-cycle treatments with AMF and without nutrient addition (Fig 2B). Belowground to aboveground biomass ratio was the highest in the 1-cycle treatment with AMF and with nutrient addition (Fig 3B). Without AMF, the number of ramets was the highest in the 1-cycle treatment irrespective of nutrient addition (Fig 2A). Leaf area of *P*. *australis* was significantly higher in the 4-cycle treatment than in the 1- or 2-cycle treatments without nutrient addition (Fig 2B). Plant height or belowground to aboveground biomass ratio did not differ between the 1- and 4-cycle treatments (Fig 3A and 3B).

## Discussion

Understanding how wetland plants respond to the presence of AMF under different levels of nutrients and drying-rewetting cycle is essential for predicting how wetland plants changes under future global change. In our study, AMF showed higher colonization rates in the 2- and 4-cycle treatments of drying-rewetting (S1 Fig), indicating that AMF survived well during the drying-rewetting cycles [15,38]. We also found that AMF promoted the number of ramets, leaf area, leaf mass and stem mass of *P*. *australis* under the 2-cycle treatment. The positive effect may not only because AMF facilitated the acquisition of limiting nutrients, but also because they enhanced the resistance of *P*. *australis* to environmental stress [23,31]. As a rhizobacteria, AMF can favor growth-promoting activity under alternate water cycles, leading to higher shoot biomass [39]. The tolerance to flooded conditions increased via 1) transpiration rates and stomatal conductance [40], and 2) increased root aeration due to increased photosynthesis from a greater allocation to aboveground growth [18]. This may explain why the stem mass, leaf mass and number of ramets were higher in the 2- and 4-cycle than in the 1-cycle treatment.

Nutrient addition can enhance plant biomass and development [41,42]. Our results demonstrated that nutrient addition had a significant positive effect on leaf mass, number of ramets, and height of *P*. *australis*, irrespective of the drying-rewetting cycles or the presence of AMF. These results were in accordance with findings of other studies with *P*. *australis* [43], *Phalaris arundinaceae* [41], and *Miscanthus sinensis* [44]. Nutrient addition, such as N, can promote aboveground growth, particularly leaf area expansion [42]. We also found that nutrient addition increased aboveground biomass and thus decreased belowground to aboveground biomass ratio of *P*. *australis*. The frequency of drying-rewetting cycles had a pronounced effect on belowground to aboveground biomass ratio of *P*. *australis* in our study. The significant positive effect on growth in the 2-cycle treatment with AMF may be attributed to the long intervals of desiccation that helped AMF colonization and promoted the growth of *P*. *australis*.

Studies showed that AMF colonization rates were significantly lower in continuously inundated conditions than in pulsed water treatments [18]. Soil desiccation facilitated infection of plants by AMF, and the duration of desiccation was greater in the 2-cycle than in the 4-cycle treatment. In addition, increasing number of drying rewetting cycles could deplete available substrates [45], which would retard plant growth. This might partly explain why significant effects were mostly detected in the 2-cycle treatment.

Previous studies show that appropriate drying-rewetting cycles could promote *P*. *australis* growth compared to persistent dry or wet conditions [46]. Our results also indicated that higher frequencies of drying-rewetting cycles did not inhibit *P*. *australis* growth, because *P*. *australis* can tolerate flooding conditions via increased root aeration due to increased photosynthesis from a greater allocation to aboveground growth [18].

In conclusion, AMF were found in the rhizospheres of *P*. *australis* in all treatments and significantly promoted *P*. *australis* growth, especially in the 2-cycle treatment. This indicated that a moderate frequency of drying-rewetting cycles may be facilitate the mutualistic symbiosis of AMF and *P*. *australis*. More ecosystem functions, such as gas emissions and carbon/nitrogen cycles, together with AMF in wetlands under global climate change should be further studied to understand the effects of global climate change on wetlands.

## Supporting information

S1 Table ANOVA results for the effects of AMF, drying-rewetting cycles, nutrient addition, and all interactions on leaf mass, stem mass, belowground mass, and total biomass of *Phragmites australis*. (DOCX)

S2 Table ANOVA results for the effects of AMF, drying-rewetting cycles, nutrient addition, and all interactions on number of ramets, leaf area, height, and below/aboveground biomass ratio of *Phragmites australis*. (DOCX)

S1 Fig Effects of AMF inoculation, drying-rewetting cycles, and nutrient addition on colonization rate of AMF on roots of *Phragmites australis*. Bars and error bars show means ± SE, respectively (n = 6).
(TIF)
