# Supplementary material for: Effects of arbuscular mycorrhizal fungi and soil nutrient addition on the growth of Phragmites australis under different drying-rewetting cycles
Source: PLoS One. 2018 Jan 29;13(1):e0191999. doi: 10.1371/journal.pone.0191999 (PMC5788386; doi:10.1371/journal.pone.0191999)
Supplement: S1 Table — (DOCX) [file pone.0191999.s001.docx]

**Supporting Information**

**S1 Table. ANOVA results for the effects of AMF, drying-rewetting cycles, nutrient addition, and all interactions on leaf mass, stem mass, belowground mass, and total biomass of *Phragmites australis*.**

|  | Leaf mass | |  | Stem mass | |  | Belowground mass | |  | Biomass | |
| --- | --- | --- | --- | --- | --- | --- | --- | --- | --- | --- | --- |
| Effect | F | *P* |  | F | *P* |  | F | *P* |  | F | *P* |
| AMF | 1.12 | 0.294 |  | 1.31 | 0.256 |  | 0.14 | 0.714 |  | 1.18 | 0.282 |
| Drying-rewetting (DW) | 1.67 | 0.198 |  | 1.74 | 0.184 |  | 0.24 | 0.785 |  | 0.73 | 0.488 |
| Nutrient addition (N) | 17.96 | **<0.001** |  | 2.85 | 0.096 |  | 0.18 | 0.674 |  | 3.11 | 0.083 |
| AMF × DW | 5.86 | **0.005** |  | 1.82 | 0.171 |  | 0.09 | 0.917 |  | 1.68 | 0.195 |
| AMF × N | 0.00 | 0.970 |  | 0.51 | 0.478 |  | 0.06 | 0.810 |  | 0.02 | 0.892 |
| DW × N | 0.09 | 0.919 |  | 0.47 | 0.628 |  | 0.35 | 0.706 |  | 0.50 | 0.610 |
| AMF × DW × N | 0.29 | 0.748 |  | 0.70 | 0.502 |  | 0.34 | 0.715 |  | 0.03 | 0.969 |

*P* values <0.05 are in bold.*{Zheng, 2015 #198;Zheng, 2015 #198}*
