# Supplementary material for: Effects of arbuscular mycorrhizal fungi and soil nutrient addition on the growth of Phragmites australis under different drying-rewetting cycles
Source: PLoS One. 2018 Jan 29;13(1):e0191999. doi: 10.1371/journal.pone.0191999 (PMC5788386; doi:10.1371/journal.pone.0191999)
Supplement: S2 Table — (DOCX) [file pone.0191999.s002.docx]

**Supporting Information**

**S2 Table. ANOVA results for the effects of AMF, drying-rewetting cycles, nutrient addition, and all interactions on number of ramets, leaf area, height, and below/aboveground biomass ratio of *Phragmites australis*.**

|  | No. of ramets | |  | Leaf area | |  | Height | |  | Below/Above | |
| --- | --- | --- | --- | --- | --- | --- | --- | --- | --- | --- | --- |
|  | F | *P* |  | F | *P* |  | F | *P* |  | F | *P* |
| AMF | 7.31 | **0.009** |  | 0.41 | 0.525 |  | 2.44 | 0.123 |  | 0.12 | 0.727 |
| Drying-rewetting (DW) | 1.21 | 0.305 |  | 1.97 | 0.149 |  | 1.92 | 0.155 |  | 1.30 | 0.281 |
| Nutrient addition (N) | 4.42 | **0.040** |  | 2.01 | 0.155 |  | 1.13 | 0.293 |  | 6.36 | **0.014** |
| AMF × DW | 3.52 | **0.036** |  | 4.48 | **0.015** |  | 0.76 | 0.472 |  | 2.94 | 0.061 |
| AMF × N | 4.42 | **0.040** |  | 2.49 | 0.120 |  | 1.18 | 0.282 |  | 0.30 | 0.586 |
| DW × N | 0.21 | 0.811 |  | 1.11 | 0.336 |  | 0.18 | 0.840 |  | 0.49 | 0.618 |
| AMF × DW × N | 0.27 | 0.764 |  | 1.71 | 0.189 |  | 1.57 | 0.217 |  | 0.88 | 0.419 |

*P* values <0.05 are in bold.
