# Supplementary figures and images for: Effects of arbuscular mycorrhizal fungi and soil nutrient addition on the growth of Phragmites australis under different drying-rewetting cycles
Source: PLoS One. 2018 Jan 29;13(1):e0191999. doi: 10.1371/journal.pone.0191999 (PMC5788386; doi:10.1371/journal.pone.0191999)

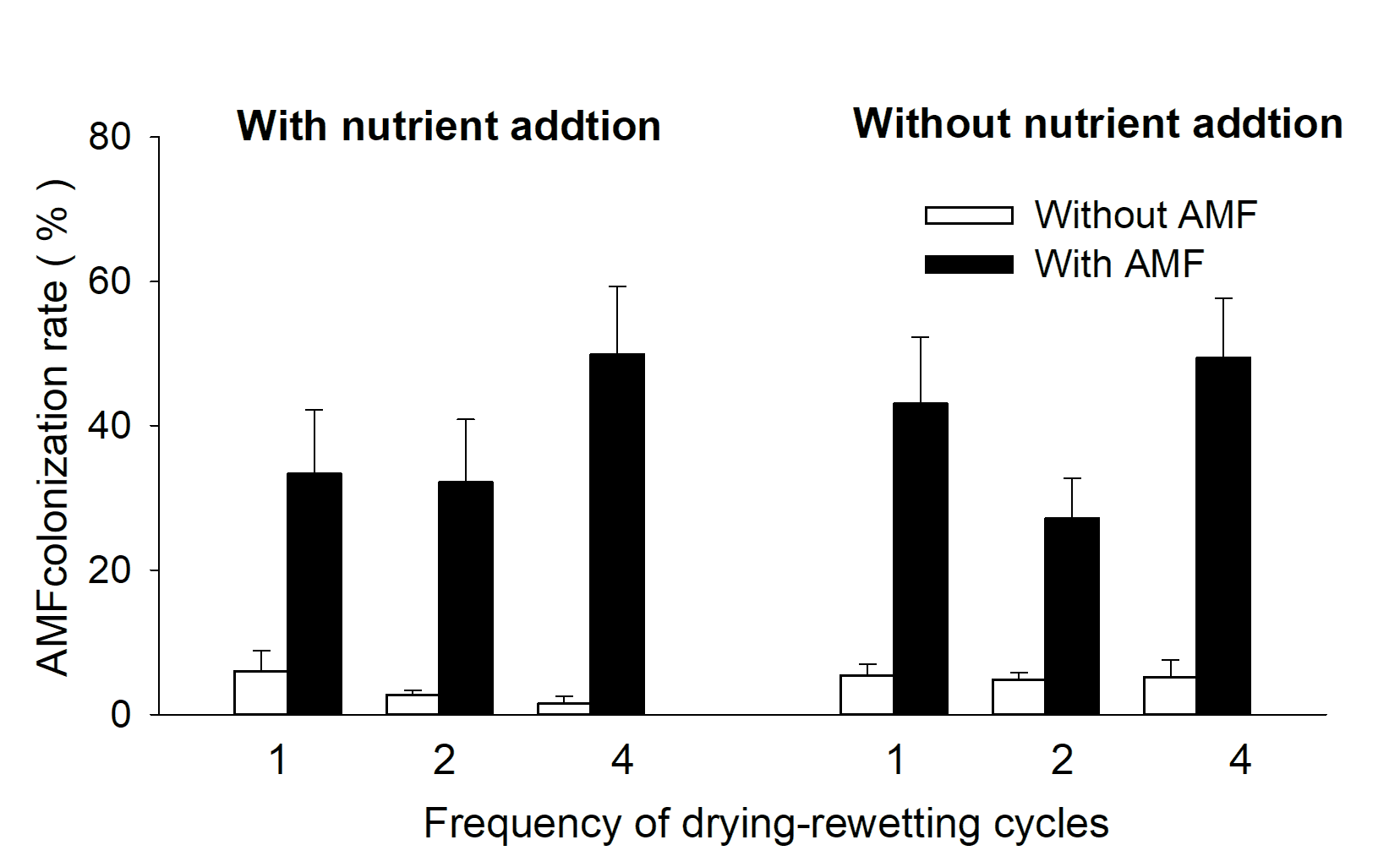

Supplement: S1 Fig — Bars and error bars show means ± SE, respectively (n = 6). (TIF) [file pone.0191999.s003.TIF]
